# Musical beauty and information compression: Complex to the ear but simple to the mind?

**DOI:** 10.1186/1756-0500-4-9

**Published:** 2011-01-20

**Authors:** Nicholas J Hudson

**Affiliations:** 1 Computational and Systems Biology, CSIRO Livestock Industries, 306 Carmody Road, St. Lucia. Brisbane, Queensland 4067, Australia

## Abstract

**Background:**

The biological origin of music, its universal appeal across human cultures and the cause of its beauty remain mysteries. For example, why is Ludwig Van Beethoven considered a musical genius but Kylie Minogue is not? Possible answers to these questions will be framed in the context of Information Theory.

**Presentation of the Hypothesis:**

The entire life-long sensory data stream of a human is enormous. The adaptive solution to this problem of scale is information compression, thought to have evolved to better handle, interpret and store sensory data. In modern humans highly sophisticated information compression is clearly manifest in philosophical, mathematical and scientific insights. For example, the Laws of Physics explain apparently complex observations with simple rules. Deep cognitive insights are reported as intrinsically satisfying, implying that at some point in evolution, the practice of successful information compression became linked to the physiological reward system. I hypothesise that the establishment of this "compression and pleasure" connection paved the way for musical appreciation, which subsequently became free (perhaps even inevitable) to emerge once audio compression had become intrinsically pleasurable *in its own right*.

**Testing the Hypothesis:**

For a range of compositions, empirically determine the relationship between the listener's pleasure and "lossless" audio compression. I hypothesise that enduring musical masterpieces will possess an interesting objective property: despite apparent complexity, they will also exhibit high compressibility.

**Implications of the Hypothesis:**

Artistic masterpieces and deep Scientific insights share the common process of data compression. Musical appreciation is a parasite on a much deeper information processing capacity. The coalescence of mathematical and musical talent in exceptional individuals has a parsimonious explanation. Musical geniuses are skilled in composing music that *appears *highly complex to the ear yet *transpires *to be highly simple to the mind. The listener's pleasure is influenced by the extent to which the auditory data can be resolved in the simplest terms possible.

## Background

"*Entia non sunt multiplicanto praeter necessitatem*." The *Lex Parsimoniae*, otherwise known as the Law of Economy or Occam's Razor (1288-1348).

"*I apologise for the length of this letter, but I didn't have time to write a shorter one*." Blaise Pascale (1623-1662).

"*Everything should be made as simple as possible, but no simpler*." Albert Einstein (1879-1955).

Succinctness is admired.

Economical arguments - made with the minimum of assumptions - are the bedrock foundation of philosophy, mathematics and science. Indeed, the highest achievements of the human intellect are widely considered to be the Laws of Physics. These Laws subsume a vast multitude of complex observations - in the case of Newton's Laws of Motion, everything from falling apples to planetary orbits - into concise, universally applicable mathematical expressions.

It appears from exploring the history of Science that the deepest insights elucidate the "*real simplicity*" that underlies the "*apparent complexity*" of a set of observations. The larger the set of observations that can be explained simply - and therefore the more succinct the level of comprehension - the more certain one feels that some fundamental "ground truth" has been unearthed. Thus, Einstein's General Relativity is considered a more fundamental theory than Newton's Universal Gravitation because it explains observations that deviate from Newton's predictions, with the minimum of extra assumptions.

In this hypothesis, I will start by briefly exploring Schmidhuber's idea [1-3] that artistic beauty shares a common cognitive process with scientific insight. That common process is the successful encoding and decoding of compressible patterns. By compression I refer to the information theoretic concept of reducing the number of bits needed to encode a given representation. What relevance does data compression have to science and art?

For science the answer is reasonably transparent. A scientific law can clearly be seen as a compression of observational data [1] (Table 1). For example, Einstein geometrized space-time. He told us that mass governs how space-time curves, while space-time governs how mass moves. In so doing, Einstein 'compressed' a host of observations (planetary orbits, the bending of light) that exist over enormous spatial scales into a single conceptual framework.

**Table 1:** Example compression algorithms from various scientific disciplines.

Subject	Compression algorithm	Originator
Philosophy	Occam's Razor	William of Occam (1288-1348)

Mathematics	Euclid's Geometry	Euclid (300 BC)

Physics	Einstein's General Relativity	Albert Einstein (1879-1955)

Chemistry	Mendeleev's Periodic Table	Dimitri Mendeleev (1834-1907)

Biology	Darwin's Evolution	Charles Darwin (1809-1882)

How might the cognitive compression abilities of someone like Einstein evolve? To answer this question - and what I believe is the related one on the origin of musical creation and appreciation - I will briefly digress into sensory biology. After all, it is our five senses that provide our direct connection to the world - and thus to both Scientific insight as well as Artistic beauty.

In determining the importance of information compression, it is useful to consider the vastness of a typical human's lifelong sensory stream. As Schmidhuber has previously pointed out [1] we live approximately 3 × 10^9 ^seconds. Encoding the entire stream of sensory information at a rate of 10^5 ^bits second^-1 ^(i.e. the demands of a film run at reasonable resolution) over this time frame results in a colossal amount of data, although not more than a human brain is capable of storing in its entirety given a reasonable set of assumptions [1]. Irrespective of the exact storage requirement, it has to be true that an effective cognitive filing and retrieval system will free up 'brain space' otherwise consumed by sensory information, thereby liberating it for competing neural processes - surely a desirable outcome.

With this information storage and retrieval problem in mind, it seems plausible that information compression primarily evolved as an economic solution geared to 1) help interpret and 2) help store the most pertinent sensory information. Successful information compression would yield an understanding of the world that was simultaneously efficient as well as useful.

## Presentation of the Hypothesis

One way to favour the realisation of adaptive behaviours - such as information compression - is to connect them to the physiological pleasure and reward centre. In sophisticated mammals this is the *nucleus accumbens *of the limbic system. With this linking between information compression and pleasure in mind, I hypothesise that information compression - originally an evolved trait to make better sense of the world - was subsequently 'parasitised' by our sensory systems. This presumably became possible - perhaps even inevitable - once successful data compression had been connected to a subjective sense of pleasure.

I contend that a seminal point in human history must have occurred when the act of compressing sensory patterns became intrinsically satisfying *in its own right*. As brain complexity and consciousness led to greater sophistication in the sensory stream's interpretation and reward system, a multitude of compressible sensory inputs could became increasingly pleasurable.

This drive for intrinsic pleasure could culminate in the emergence of music and poetry for compressible sound, and sculpture and painting for compressible sight. Thus, I hypothesise that the evolution of pleasurable information compression paved the way for not only philosophy, mathematics and science but also art, music and sculpture, *sensu *[1].

To provide the conceptual foundation for this hypothesis I will briefly explore the existing evidence for a link between information compression and musical beauty. I will focus my analysis and discussion primarily on music because 1) the enigmatic nature of its origin has been the subject of much recent research and debate [1,4-14] 2) because it transcends cultures and 3) because it yields well to mathematical analysis [4,8]. However, as Schmidhuber has pointed out [1-3], the compression principle is deep enough to apply well to other art forms.

My hypothesis builds on Schmidhuber's insights by 1) its particular focus on music 2) the intriguing possibility that enduring musical masterpieces are "losslessly" more compressible than other "less sophisticated" pieces (that is, the most beautiful music has low Kolmogorov complexity despite initial perceptions of apparent high complexity) and 3) by framing the origin of the compression algorithm in the context of a possible parsimonious evolutionary sequence, thereby grounding it in biology.

### Information Theory and Data Compression

This principle - deceptively simple rules explaining apparently complex data - can be defined and explored within the framework of Information Theory. This is not a new concept, having been thoroughly explored by Schmidhuber [1] among others. Within this information theoretic context, data compression - otherwise known as source coding - is the process of encoding information using fewer bits than the original unencoded representation; a bit referring to the fundamental unit of information.

Information has a specific meaning in Information Theory. Thus, when comparing an encyclopaedia to a random sequence of letters of the same length, from our perspective as human consumers the encyclopaedia contains more 'useful information.' Yet from an Information Theory perspective it actually contains less total information because regularities and patterns in the data make it more compressible.

There are a number of methods for understanding and quantifying complexity within an Information Theory framework. The Minimum Description Length Principle is a formalisation of Occam's Razor in which the best hypothesis for a given set of data is the one that leads to the largest compression of the data [15]. The fundamental idea being that any repeating patterns in the data can be exploited to compress it. The length of the shortest program that outputs the data is called the Kolmogorov Complexity, the Descriptive Complexity or the Algorithmic Entropy.

A few simple examples suffice to illustrate the principle. The regular data stream "10101010101010101010" can be easily compressed to "10(10 times)." On the other hand, a truly random sequence of numbers, say "57622390136573928476" is barely compressible at all, and has to be described in full. Meanwhile, the enigmatic Π ("3.1415926535897932384"), an irrational number comprising an infinite - apparently random - stream of digits, actually contains only a few bits of information because a short program can fully recreate it. Thus, Π possesses the interesting conceptual property of being 'apparently' complex but 'really' simple. I believe this same dual property lies at the heart of artistic as well as scientific beauty. The rest of the hypothesis will explore the evidence for this proposition.

### Lossless versus Lossy compression

In Information Theory there are two broad forms of data compression, "*lossless*" and "*lossy*." Lossless compression algorithms exploit statistical redundancy thereby retaining the entire information content of the message faithfully despite using fewer bits of information. Einstein's quote ("*things should be made as simple as possible, but no simpler*") is a fine working definition of Lossless compression, and reciprocally, lossless compression is a fine ultimate goal of science.

On the other hand, Lossy compression algorithms reduce information content via "acceptable" losses in fidelity. What is considered "acceptable" is subjective. It may depend on the intended use of the message and the opinion of the receiver. Lossy compression is certainly common in the visual Arts where the basic concept of a complex 3D object can be clearly, but not perfectly, represented by relatively few lines. Between December 5th 1945 and January 17^th ^1946, Pablo Picasso famously explored the extent to which a bull could be "lossy compressed" through visual art (refer to [16]), although in conveying the 'essence' of a bull it is doubtful he explicitly considered his work in formal information theoretic terms.

During information transfer, compression refers to the process than encodes the original representation using fewer bits of information, and decompression refers to the decoding process used to recreate the original representation.

### Pattern recognition

**Figure 1:**
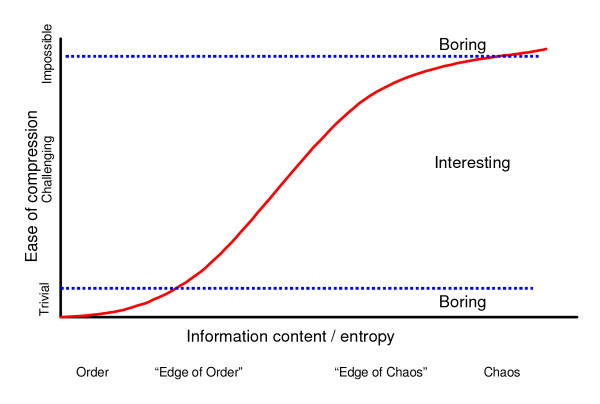
**From a compression standpoint, highly ordered patterns are boring because they are too simple while random chaotic patterns are boring because they are too complex**. On the other hand, intermediately complex patterns - those that promise a chance of compression following some effort - are of particular interest. I use the terms the "*edge of order*" and the "*edge of chaos*" to define points of inflection in the imagined relationship between information content and ease of compressibility. The high pay-off zone is somewhere in the middle.

We understand the world through patterns. However, not all patterns are born equal. I will argue the case that we find particularly pleasurable those patterns that are neither too simple nor too complex, *sensu *[17]. There is little point in encouraging the resolution of problems that are either trivial or insoluble. It seems plausible that evolution would reward the solution of high pay-off problems that are challenging but soluble, and achieve this by endowing them with a particularly strong sense of pleasure. The relationship between these parameters may take the form I have represented schematically in Figure 1. I borrowed the phrases "*The Edge of Order*" and "*The Edge of Chaos*" from [18].

Given that compression ability likely varies between individuals, across development and based on experience, the location of the computational 'sweet spot' is elusive. This highlights the extent to which even an 'objective' measure of beauty can still manifest in a manner suggestive of subjectivity.

### Competing hypotheses on the Biological Origin of Music

All cultures make music, though no one knows why; it is not obviously useful in the way cooking or language are [4]. Thus, the origin of music continues to mystify scientists. According to [7] throughout human history, on every part of the globe, in every extinct and extant culture, individuals have played and enjoyed music. According to Oliver Sacks we turn to music because of its ability to move us and induce states of mind - and that we have all had the experience of being transported by the sheer beauty of music [19]. Arguably the most intriguing question about music concerns its evolutionary origins: how do we reconcile its cross-cultural ubiquity on the one hand, with a lack of a clear adaptive story on the other?

Of the evolutionary hypotheses that have been posited, some emphasise a deep relationship between music and language [6,7]. Alternatives include Pinker's "cheesecake hypothesis" [20], Darwin's sexual selection hypothesis [21], Dunbar's group "grooming hypothesis" [5,22], Storr's social cohesion hypothesis [23] and Trehub's caregiving model [12,13]. Other evolutionary possibilities, reviewed in [24] include perceptual development, motor skill development, conflict reduction, safe time passing and trans-generational communication.

Here, I subscribe to Schmidhuber's Theory of Creativity [1], which unifies a range of artistic and scientific cognitive processes with the information theoretic concept of data compression. Links between beauty and information theory have also been explored by Abraham Moles and Frieder Nake [25,26]. These viewpoints are broadly in line with the philosopher and mathematician Alfred North Whitehead who claimed "*Art is the imposing of a pattern on experience and our aesthetic enjoyment is recognition of the pattern*" [27].

The intense degree of pleasure associated with listening to music is a mystery closely related, in my view, to its biological origin [11]. According to [11] there are no direct functional similarities between music and other pleasure-producing stimuli: it has no clearly established biological value (cf food, love, sex), no tangible basis (cf. pharmacological drugs and monetary rewards), and no known addictive properties (cf gambling and nicotine). Having said this, some very recent progress has been made into identifying the organic basis of musical appreciation. Using Positron Emission Tomography, [28] discovered that minor consonant chords activate the right striatum (reward and emotion) whereas major consonant cords activate the left middle temporal gyrus (orderly information processing).

### Caveat

Before I explore the relationship between information compression and musical beauty in more detail I wish to head off a source of possible confusion. Music (and indeed other Arts) can have an 'extrinsic' emotional appeal entirely separate from what I view as its 'intrinsic' cognitive value. This is by 1) representing a certain sub-culture or belief system that the receiver strongly relates to, for example female submissiveness and male violence in hip hop music and/or 2) stimulating the receiver through historical association.

For this hypothesis I am exclusively interested in a particular aspect of intrinsic cognitive value - that is, the pleasure derived from appreciating the information contained in the art form. Clearly, there are other intrinsic influences on musical beauty - such as rhythm, pitch and timbre - but these have been purposefully ignored to simplify exposition of the hypothesis.

### Musical Patterns

Music is clearly full of patterns. Some patterns relate to harmony, the vertical stacking of notes - and some to melody, the horizontal spacing of notes. The most delightful compositions balance predictability and surprise [8]. This appreciation "....*rests on our ability to discern patterns in the notes and rhythms and use them to make predictions about what will come next. When our anticipations are violated, we experience tension; when the expectation is met, we have a pleasurable sense of release*" [4].

### Is beautiful music highly compressible?

Schmidhuber's Theory of Creativity states that beautiful Art is influenced by the extent to which unexpected information compression progress is possible [1]. This Theory builds on an earlier paper which outlined the appeal of low Kolmogorov complexity visual Art [2]. For example, drawings utilising - although not in any immediately apparent way - basic geometric shapes look appealing [2]. I am interested in the power of these insights to elucidate the biological origin of music and shed light on the nature of its beauty. Therefore, to seek confirmation of Schmidhuber's hypothesis in the context of music, I elected to compare the ability of Lossless compression algorithms to compress different pieces of music; a concept previously voiced, but not explored, by [9].

Ranking musical compositions by beauty is clearly a task fraught with issues of subjectivity. Nevertheless, I believe it to be the case that most reasonable people would accept Ludwig Van Beethoven to be a greater musical genius than, say, Kylie Minogue. But what is it about Beethoven's Art that supports such a viewpoint?

At some level it must reflect a prevailing belief that his music is more beautiful than Kylie Minogue's. With this view in mind, one can make a baseline assumption that a Beethoven Symphony represents a higher level of beauty than a range of "less sophisticated" compositions. Along these lines, I was interested to see whether enduring musical masterpieces, such as Beethoven's Symphonies, might be more compressible than other musical compositions.

As a small initial first step towards this goal, I examined a web page where comparisons had already been made in the ability of a range of lossless compression algorithms to compress various test audio files [29]. The purpose of the website was not a theory of musical beauty, but rather a practical exploration of compression algorithms in a range of circumstances. In brief, a range of lossless algorithms (Waveform Archiver, LPAC, Audiozip, Monkey's Audio and RKAU) were run on musical compositions from the following genres: Classical, Techno, Rock, Pop, and random noise. (A caveat: the five compression algorithms assessed were discovered [29] to produce higher rates of compression than other programs, although that does not imply they are universally better. Different algorithms work best on different kinds of music.)

The smallest file size was determined in megabytes and expressed as a percentage of the original file size. Intriguingly, based on these (albeit very limited) pilot data it does appear to be the case that the representative compositions from Pop, Rock and Techno music are less compressible than Choral and Orchestral masterpieces. Pink noise stereo representing random noise, was highly information-rich as expected, being compressible to only 85.8% of original.

For example, Beethoven's 3^rd ^Symphony was strongly compressible to only 40.6% of the original file size, whereas the Techno piece "*Theme from Bubbleman" *by Andy Van, the Pop piece "*I should be so Lucky*" by Kylie Minogue and the Rock piece "*White Wedding*" by Billy Idol were considerably less compressible, compressing to 68.5%, 69.5% and 57.5% of original file size respectively. Therefore, Beethoven's 3^rd ^Symphony is a better example of low Kolmogorov complexity Art [2] than Kylie Minogue's "*I should be so Lucky*."

But there is a further interesting observation. The relatively low compressibility of the Pop pieces is at odds - at least with my perception - that they *appear *on the surface to be simpler and more ordered than their Classical counterparts. Furthermore, the disparity cannot easily be attributed to the presence or absence of human vocals. Gothic Voices version of Hildegard von Bingen's 12^th ^century choral masterpiece *Columbia aspexit *compresses very strongly to 34.7%.

Therefore, a surprising feature of Beethoven's 3^rd ^symphony is that - somewhat analogous to the numerical properties of Π - despite having a very short algorithmic description in reality, it appears on initial perception to have a very long algorithmic description.

One might say - at least from an information theoretic perspective - that Classical music is apparently complex but really simple, while Popular music is apparently simple but really complex.

The lasting impression that Classical masterpieces have had on human culture, and the high esteem that composers such as Bach, Beethoven and Mozart are held in, may reflect an intrinsic appreciation for successful information compression that is held below our conscious awareness.

I speculate that when we appreciate music, a major influencing factor is the release of pleasure that comes from performing a surprisingly profound audio data compression. By this logic, one would anticipate the level of pleasure to scale with the mismatch between the apparent complexity initially perceived by our ears and the real simplicity subsequently resolved in our minds.

This overall compression 'epiphany' is more dramatic in Classical masterpieces because the extent of the mismatch - or put another way, the magnitude of the successful information compression - is that much higher, and therefore our sense of pleasure that much more acute. This argument exactly mirrors Schmidhuber's concept of compression progress influencing individual perception of beauty [1].

**Figure 2:**
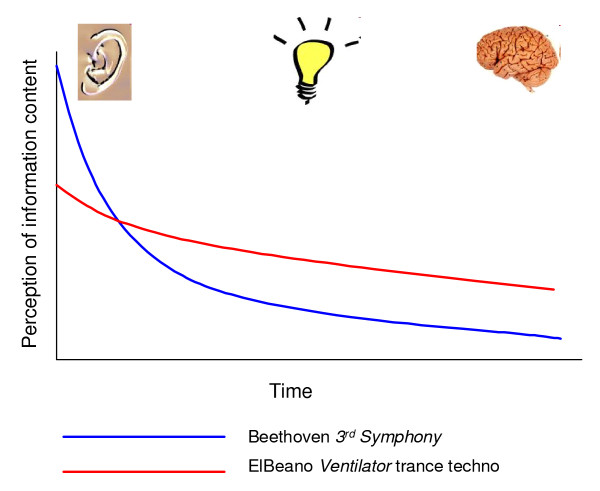
**The appreciation of music is a function of information compression**. From our perspective as human listeners, this reflects the mismatch in complexity between what our ears initially perceive, versus what our brains ultimately interpret. This hypothesis is schematically represented here for two pieces of music: Beethoven's 3^rd ^Symphony and ElBeano's Ventilator trance techno. The two compositions are approximately the same length (4 minutes 7 seconds versus 4 minutes 9 seconds), and approximately the same initial audio file size (43.6 Mb versus 44.0 Mb). Beethoven's piece has the interesting dual property of *appearing *more sophisticated but *being *more simple (compressing to 40.6% as compared to 65.8% of original file size). The learning curve, or "information compression epiphany", is thus substantially larger and more rewarding for Beethoven's piece.

The mis-match between perceptual complexity and cognitive simplicity is schematically illustrated for two musical pieces of similar length and original file size, Beethoven's 3^rd ^Symphony and ElBeano's Ventilator trance techno. These two pieces compress to very different extents (Figure 2). My personal perception is that Beethoven's 3^rd ^Symphony sounds more sophisticated (complex?) than ELBeano's Ventilator trance techno, and yet it actually compresses *more *strongly. It therefore must be the case that Beethoven's piece contains more information regularities, but the skill and subtlety with which they are woven into the composition makes them *less *readily apparent. The simplicity of their message - as reflected by compressed file size - only yields on repeated listenings. This learning curve - or compression progress [1] - may explain the phenomenon of a piece of music "growing on us" over time.

Listening to enduring Classical music elicits such a strong sense of pleasure for most listeners because their information complexity is cleverly situated in the computational sweet-spot; that is, the compositions are neither so simple that they are trivial to compress nor so complex that they are impossible to compress. Like all the best puzzles, they are challenging but doable. If Politics is the Art of the Possible, and Science the Art of the Soluble [30], then Music may be the Art of the Compressible.

Possible locations of various musical compositions in terms of information complexity are given in Table 2 along with some examples from other art forms.

**Table 2:** Information compression and the Arts.

REGULARITY (COMPRESSIBILITY)	MUSIC	VISUAL ARTS	POETRY	EMOTIONAL RESPONSE
HIGHLY REGULAR (trivial compression)	Repeated notes, simple ascending scales	Simple geometric shapes	Limericks, nursery rhymes (anapaestic tetrameter)*	Indifference, boredom (irritation?)

EDGE OF REGULARITY (challenging compression)	Beethoven (Classical)	Picasso (Classicism, Surrealism, Cubism)	Shakespeare (Iambic pentameter)	Pleasure

EDGE OF CHAOS (very challenging compression)	Duke Ellington (Jazz)	Jackson Pollack (Abstract expressionism)	Robert Louis Stevenson (Free verse)	Pleasure

CHAOTIC (impossible compression)	Noise	Random splashes of paint	Random letter/word generator	Indifference, boredom (irritation?)

The most appealing Artistic patterns are in between order and chaos, with those that are challenging but highly compressible including Beethoven's music. * Limericks and nursery rhymes arguably sit closer to the "edge of regularity" than "highly regular", but were positioned here to discriminate them from more sophisticated literary art.

By this hypothesis, it is not low Kolmogorov complexity *per se *that is a feature of musical beauty, but rather the mismatch between how much information a piece *appears *to contain on first hearing, versus how much information it *actually *contains once the data has been compressed. One might say that enduring Artistic masterpieces possess 'concealed' low Kolmogorov complexity - and thus entice us with the promise of what has been termed 'compression progress' [1] only after sustained effort.

## Testing the Hypothesis

How might the "audio compression" hypothesis be put to a rigorous test. First of all, it is imperative that a formal, exhaustive and statistically robust comparison of different musical compositions is undertaken and matched against some measure of subjective human pleasure, perhaps using the data outlined in [11] as a test set. The analysis would be strengthened by a wide range of compression algorithms. The output will obviously highlight individual human variation in taste, but may also allow the detection of an additional signal relating pleasure to information compression. Of particular interest will be whether the most enduring and beautiful pieces, from all musical genres, relate to those that are subjectively perceived as being complex but turn out to be highly compressible in practice.

It will also be important to establish the extent to which the compressibility of the different musical compositions of a given duration reflects differences in 1) amount of silence versus *bona fide *differences in the statistical redundancy present in the melodies and harmonies themselves and 2) differences in overall tempo, as *presto *pieces will contain more information than *adagio *pieces, all else being equal.

At this juncture, it is appropriate to flag an important distinction between compressibility versus *change *in compressibility. On the one hand, running a formal compression algorithm on an audio file provides an 'objective' (notwithstanding the stated limitations) measure of pure 'compressibility.' However, on the other hand the subjective perception of a human listener, and their ability to compress the music cognitively, may change over time and with experience, including experience with that particular piece of music. Thus, the human sensation of pleasure that we are trying to explain may well be influenced by a perception of a *change *in compressibility, as opposed to just compressibility.

Along these lines, it is not clear whether the subjective experience of music growing on us over time represents 1) a pre-existing cognitive algorithm whose compression potential is only gradually accessed, or 2) an entirely new compression algorithm developed through the challenge and experience of understanding that particular piece of music.

The argument I have presented partly rests on accepting my subjective assessment that Beethoven's 3^rd ^symphony is initially perceived as being more complex than ELBeano's Trance Techno. Although beyond the scope of the present Hypothesis, this argument could be formalised via Schmidhuber's "before-and-after effect", and interested readers are directed to his research in this area.

One further means of trying to get a handle on compression progress and its impact on musical appreciation could be through the field of artificial intelligence. For example, recurrent neural networks can 'learn' to improve their problem solving ability e.g. [31]. The compression progress achieved by recurrent neural networks could be assessed on particular pieces of music and related back to the subjective quality attached to those pieces by human listeners. The relevance of this approach would depend on the extent to which the behaviour of the artificial recurrent neural network resembled the cognitive performance of a real human brain.

### The biological origin of the compression and pleasure connection

Accounts of major scientific breakthroughs (i.e. powerful and novel information compressions) clearly suggest that insight is rewarded by a visceral thrill. Consider the following quote from Garett Lisi following his discovery of the proposal for an E8-based unification model for all the particles and physical forces "...*my mind exploded with the implications and the beauty*..." [32].

It appears to me from reading this account, and many others like it, that much of the pleasure associated with a scientific breakthrough is largely intrinsic ("...*the beauty*"). That is, it relates to the satisfaction associated with a successful computation, rather than being extrinsic (a potential award ceremony in Stockholm). Why is scientific insight accompanied by a thrill?

### Science first, Art second

This hypothesis for the evolutionary origin of music composition and appreciation is predicated on a pre-existing connection between pleasure and successful information compression. This defers the question to why might this link have evolved in the first place? As Schmidhuber [1] points out, the concept of daylight is a useful compression of the repeated observation that the sun rises regularly every morning. This sort of compression ability would presumably underpin a more manageable and predictive understanding of the environment, thereby increasing fitness relative to rivals with poorer cognitive performance, and thus being potentially subject to selection.

Compression clearly forms the foundation of science. After all, scientific insight tends to relate - admittedly at various levels of abstraction - to some sort of predictive understanding of the environment, where environment can mean something as little as a sub-atomic quantum state or as large as the entire universe. The most profound scientific insights (Universal gravitation, General Relativity and so on) compress vast numbers of apparently diverse environmental observations into concise Laws that can sometimes be expressed using nothing more than a handful of symbols.

Obviously, adaptive data compressions may not always be held in our conscious awareness, but that is beside the point. All that is required is that the successful compression process is rewarded physiologically. Once the compression and pleasure connection has been forged by evolution, it opens up the possibility for compressing all sorts of subsequent sensory information sources. That is, the joy of compression can then be pursued for its own sake, simply for the release of intrinsic pleasure associated with the process. Skill at information compression is a parsimonious explanation for the coalescence of musical and mathematical talent sometimes observed in some exceptional individuals. The sub-conscious nature of the information appraisal is quite consistent with our difficulty in clearly articulating *why *we find a piece of music beautiful, even though we *know *it sounds beautiful.

### The Encoder and the Decoder in Art and Science

In information theory, the compression process involves an encoding step performed by the sender, and a decompression step performed by the receiver. From a certain perspective it can be seen that Art and Science differ fundamentally.

In Art the composer performs the compression (e.g. from landscape to painting) and the viewer or listener performs the decompression (e.g. from painting to landscape). In Science the Laws (information compressions) that govern the Universe's behaviour pre-exist, leaving the scientist with 'only' the task of reverse-engineering them. This reverse-engineering emerges from cognitive processes that sift through the sensory data.

The path to the most profound scientific compressions seems to depend on the cognitive style of the individual scientist, there does not appear to be a unique winning recipe. While the end result is a profound information compression in all cases, the actual cognitive exploration that yields the insight seems to differ widely. According to Nambu [33] the cognitive style of eminent theoretical physicists falls predominantly into one of 3 major styles: heuristic, bottom-up and inductive (e.g. Heisenberg), axiomatic, top-down and deductive (e.g. Einstein), or abstract, revolutionary and aesthetic (e.g. Dirac).

In any case, there is a sense in which artists are fortunate; they get to *create *the potential for compression, while scientists merely *discover *the potential for compression.

A different perspective, emphasising the commonalities between Artists and Scientists can be found in [1]. This argues that Scientists invent experiments to create data that allows for further compression. This may be true, but unless the Law has some external *reality *it will not be discovered, no matter what set of experiments is undertaken. Thus, it appears to me that there is a limitation imposed on the compression progress a Scientist can make that does not exist for the Artist.

## Implications of the Hypothesis

This hypothesis, if supported by the recommended experiments, will shed new light on the open question as to the biological origin of music. Musical appreciation may be influenced by a deep cognitive process relating to information compression. Musical beauty may have a more objective basis than is commonly accepted, relating to the complexity mis-match between initial sensory perception and ultimate cognitive resolution. Musical masterpieces may share an information compression property that transcends composer, era, instrument and style. Musical geniuses are skilled at composing stimulating auditory data that possesses deceptively low Kolmogorov complexity. The link between mathematically and musically-talented individuals may have a simple, parsimonious explanation relating to the exercise of a single cognitive skill. Information Theory may help unite the Two Cultures [34] of Art and Science.

### Final Conclusion

As with all generalisations, a frank discussion of the presence of both supporting examples and counter-examples will illuminate where and why the musical information compression hypothesis breaks down.

With this caveat borne in mind, I contend that musical beauty - like the deepest scientific and mathematical insights - is that which according to our senses is *apparently *complex but according to our minds is *really *simple.

## Competing interests

The author declares that he has no competing interests.
